# Corynoline Isolated from *Corydalis bungeana* Turcz. Exhibits Anti-Inflammatory Effects via Modulation of Nfr2 and MAPKs

**DOI:** 10.3390/molecules21080975

**Published:** 2016-07-27

**Authors:** Chunjuan Yang, Chengyue Zhang, Zhibin Wang, Zhenqiu Tang, Haixue Kuang, Ah-Ng Tony Kong

**Affiliations:** 1 Department of Pharmaceutical Analysis and Analytical Chemistry, College of Pharmacy, Harbin Medical University, 157 Baojian Road, Nangang District, Harbin 150081, China; 2 Center for Phytochemical Epigenome Studies, Department of Pharmaceutics, Ernest Mario School of Pharmacy, Rutgers, The State University of New Jersey, Piscataway, NJ 08854, USA; chengyue.zhang@gmail.com (C.Z.); wzbmailbox@126.com (Z.W.); 3 Key Laboratory of Chinese Materia Medica (Ministry of Education), Heilongjiang University of Chinese Medicine, 24 Heping Road, Xiangfang District, Harbin 150040, China; 13199472861@163.com (Z.T.); hxkuang@yahoo.com (H.K.)

**Keywords:** corynoline, anti-inflammation, Nrf2, MAPKs

## Abstract

*Corydalis bungeana* Turcz. is an anti-inflammatory medicinal herb used widely in traditional Chinese medicine for upper respiratory tract infections. It is demonstrated that corynoline is its active anti-inflammatory component. The nuclear factor-erythroid-2-related factor 2 (Nrf2)/antioxidant response element (ARE) pathway and the mitogen-activated protein kinase (MAPK) pathway play important roles in the regulation of inflammation. In this study, we investigated the potential anti-inflammatory mechanism of corynoline through modulation of Nfr2 and MAPKs. Lipopolysaccharide (LPS)-activated RAW264.7 cells were used to explore modulatory role of NO production and the activation of signaling proteins and transcription factors using nitrite assay, Western bloting and qPCR. Treatment with corynoline reduced production of nitric oxide (NO) and the protein and mRNA levels of inducible nitric oxide (iNOS) and cyclooxygenase-2 (COX-2) Treatment also significantly increased the expression of Nrf2, quinone oxidoreductase 1 (NQO1) and hemeoxygenase-1 (HO-1) at the mRNA and protein levels, which demonstrated that corynoline may protect cells from inflammation through the Nrf2/ARE pathway In addition, corynoline suppressed the expression of inflammatory cytokines, such as tumor necrosis factor-α (TNF-α) and interleukin-1β (IL-1β), at the mRNA and protein levels. Furthermore, molecular data revealed that corynoline inhibited lipopolysaccharide-stimulated phosphorylation of c-jun NH2-terminal kinase (JNK) and p38. Taken together, these results suggest that corynoline reduces the levels of pro-inflammatory mediators, such as iNOS, COX-2, TNF-α and IL-1β, by suppressing extracellular signal-regulated kinase 1/2 (ERK) and p38 phosphorylation in RAW264.7 cells, which is regulated by the Nrf2/ARE pathway. These findings reveal part of the molecular basis for the anti-inflammatory properties of corynoline.

## 1. Introduction

Inflammation is a physiological defense response of the body to tissue damage caused by microbial pathogen infections, chemical irritation and/or wounding [1]. There are two types of inflammatory responses: acute inflammation and chronic inflammation. An acute inflammatory the response is usually beneficial because it is a part of the defense response to irritation, injury and infection, and is characterized by pain, redness, swelling and sometimes loss of function. However, failure to resolve acute inflammation may lead to chronic inflammation and various diseases, including cancer [2]. Chronic inflammation has been identified in various steps of tumorigenesis, including cellular transformation, promotion, survival, proliferation, invasion, angiogenesis and metastasis [3,4]. TNF-α and IL-1β are two pro-inflammatory mediators that are primarily produced by activated macrophage cells and contribute to the course of many inflammatory diseases. Moreover, several studies have reported increased levels of these cytokines in some types of cancer, thereby providing strong support for their possible roles in cancer progression [5,6].

Lipopolysaccharide (LPS) is an endotoxin found in the outer membrane of Gram-negative bacteria. LPS elicits a strong immune response by stimulating macrophages to produce pro-inflammatory cytokines such as TNF-α and IL-1β, as well as other inflammatory mediators such as NO, iNOS and COX-2 [7,8,9,10]. The signal transduction and expression of iNOS and COX-2 in response to the LPS involve complex processes [11,12]. MAPKs, a group of signaling molecules, play critical roles in the regulation of cell growth and differentiation and are also responsible for transcriptional regulation of COX-2 and iNOS [13,14]. As the first line of host defense against bacterial infections and cancer growth, macrophages play an important role in the initiation of adaptive immune responses. Macrophages release several inflammatory cytokines, such as IL-1β, IL-6 and TNF-α, following stimulation with LPS, all of which directly induce the tumoricidal or inflammatory activity of macrophages [15]. Thus, RAW264.7 cells, a macrophage-like cell line, are considered a suitable model for screening and evaluating candidate drugs that possess anti-inflammatory potential.

Oxidative stress is also an important inducer of inflammation because it activates the redox-sensitive pro-inflammatory signaling pathway [16]. Nrf2 is ubiquitously expressed in a wide range of tissue and cell types and plays a critical role in the regulation of inflammation because the regulation of ROS production is closely related to anti-inflammatory processes [17,18].

Nowadays, the commercially approved anti-inflammatory drugs are effective for the relief of the main inflammatory symptoms. However, most of them are inadequate for chronic use. The recent and emerging scientific community slant is to the herbal medicines that could represent a treasure for the discovery of new active compounds and for the development of new drugs and potentially useful therapeutic agents [19]. The anti-inflammatory functions of these natural extracts were the key role of follow-up phytochemical and pharmacological studies that led to the identification and characterisation of a variety of natural active compounds [20]. The genus *Corydalis* (family *Fumariaceae*) comprises 470 species. *Corydalis*, which has many pharmacological activities, is native to China, the Himalayas of Nepal, Pakistan and India, and also found in mountainous regions of Eastern Africa [21]. Govaniadine, an alkaloid isolated from *Corydalis govaniana* Wall. It is reported that the peripheral and central analgesic effects of govaniadine could be in part related to the involvement of COX-2 activity and by its interaction with the opioid system [22]. *Corydalis bungeana* Turcz. (CB) is a perennial herb containing several pharmacologically important alkaloids such as corydaline, 12-hydroxycorynoline, protopine, acetylcorynoline, and corynoline [23]. The dried whole plant is referred in traditional Chinese medicine as Herba Corydalis Bungeanae, and is used for clearing heat and toxins, as well as an anti-inflammatory [24]. CB has been used for treating influenza, infections of the upper respiratory tract, bronchitis, tonsillitis, acute nephritis, and pyelonephritis [25]. Corynoline is the major alkaloid component derived from CB, which contribute to the anti-inflammatory effects of the alkaloid extract of CB [26]. However, its molecular targets and the mechanisms underlying its anti-inflammatory activities are still poorly defined. In the present study, we evaluated the Nrf2/ARE activation activity and anti-inflammatory potential of corynoline in ARE-luciferase-transfected HepG2-C8 cells and in LPS-induced RAW264.7 murine macrophages, respectively, to clarify these mechanisms.

## 2. Results

### 2.1. Corynoline, Acetylcorynoline and Protopine Induce Transcriptional Activation of ARE-Luciferase

**Figure 1:**
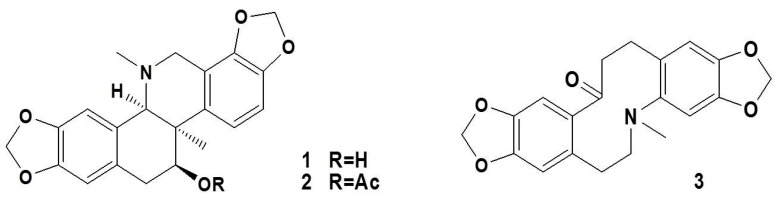
The structures of corynoline (**1**), acetylcorynoline (**2**), and protopine (**3**).

**Figure 2:**
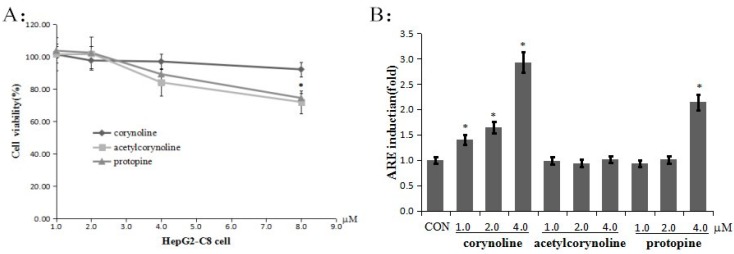
Corynoline, acetylcorynoline and protopine inhibit cell growth and induce ARE activity in HepG2-C8-ARE luciferase cells. (**A**) Inhibition of cell growth by corynoline, acetylcorynoline and protopine. For these experiments, HepG2-C8-ARE luciferase cells were seeded onto a 96-well plate and incubated with different concentrations of corynoline, acetylcorynoline or protopine or with DMSO as the vehicle control, for 24 h. MTS reagent was added to each well, and the absorbance of the formazan product was read at 490 nm; (**B**) Induction of ARE activity in HepG2-C8-ARE luciferase cells. The human hepatoma HepG2-C8-ARE luciferase cells were seeded onto a 96-well plate and treated with different concentrations (1, 2 or 4 μM) of corynoline, acetylcorynoline or protopine for 24 h. The results are expressed as the mean ± SE; * *p* < 0.05 compared with the vehicle group.

The MTS assay was used to determine the cytotoxicity of corynoline, acetylcorynoline and protopine (Figure 1) in HepG2 cells after 24 h of treatment. None of the compounds displayed significant toxicity at concentrations of up to 4 μM (Figure 2A). Therefore, these concentrations were used for the subsequent studies. As oxidative stress is an important mediator of inflammation, the corynoline-, acetylcorynoline- and protopine-induced transcriptional activation of ARE was evaluated. HepG2-C8-ARE luciferase cells were treated with various concentrations (1, 2 or 4 μM) of corynoline, acetylcorynoline or protopine for 24 h, and ARE induction was measured using a luciferase assay. The results demonstrated that corynoline and protopine increased ARE induction to different degrees. Of these compounds, the corynoline antioxidant capacity was the strongest (Figure 2B).

### 2.2. Corynoline, Acetylcorynoline and Protopine Inhibit NO Production in LPS-Induced RAW264.7 Cells

**Figure 3:**
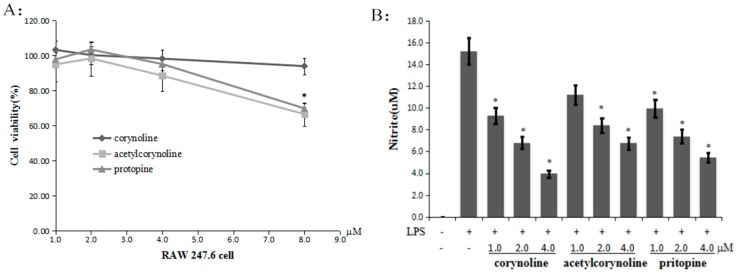
Corynoline, acetylcorynoline and protopine inhibit cell growth and LPS-induced NO production in Raw264.7 cells. (**A**) Inhibition of cell growth by corynoline, acetylcorynoline and protopine. For these experiments, RAW264.7 cells were seeded onto a 96-well plate and were incubated with different concentrations of corynoline, acetylcorynoline or protopine or with DMSO as the vehicle control, for 24 h. MTS reagent was added to each well, and the absorbance of the formazan product was read at 490 nm; (**B**) Inhibition of LPS-induced NO production by corynoline, acetylcorynoline and protopine. RAW264.7 cells were seeded onto a 96-well plate and treated with different concentrations of corynoline, acetylcorynoline or protopine with LPS (150 ng/mL) for 24 h. The isolated supernatant fractions were mixed with an equal volume of Griess reagent and incubated at room temperature for 10 min. Nitrite production was measured by reading the absorbance at 540 nm. Each point represents the mean ± SE; * *p* < 0.05 compared with the LPS-treated group.

The toxicity of these compounds was observed in cells treated with LPS in combination with corynoline, acetylcorynoline or protopine. As demonstrated by the MTS assay, 4 μM corynoline, acetylcorynoline or protopine did not induce significant cytotoxicity (Figure 3A). Therefore, these concentrations were used for subsequent studies. To evaluate the inflammatory effect of corynoline, acetylcorynoline and protopine, we examined nitrite levels to investigate their inhibitory effects on NO production in LPS-induced RAW264.7 cells. As illustrated in Figure 3B, corynoline, acetylcorynoline and protopine all significantly reduced LPS-induced NO production in a dose-dependent manner. Of these compounds, corynoline exhibited the strongest inhibition of LPS-induced NO production.

### 2.3. Corynoline Up-Regulates the Expression of Nrf2, HO-1 and NQO1 at Both the mRNA and Protein Levels in LPS-Induced RAW264.7 Cells

**Figure 4:**
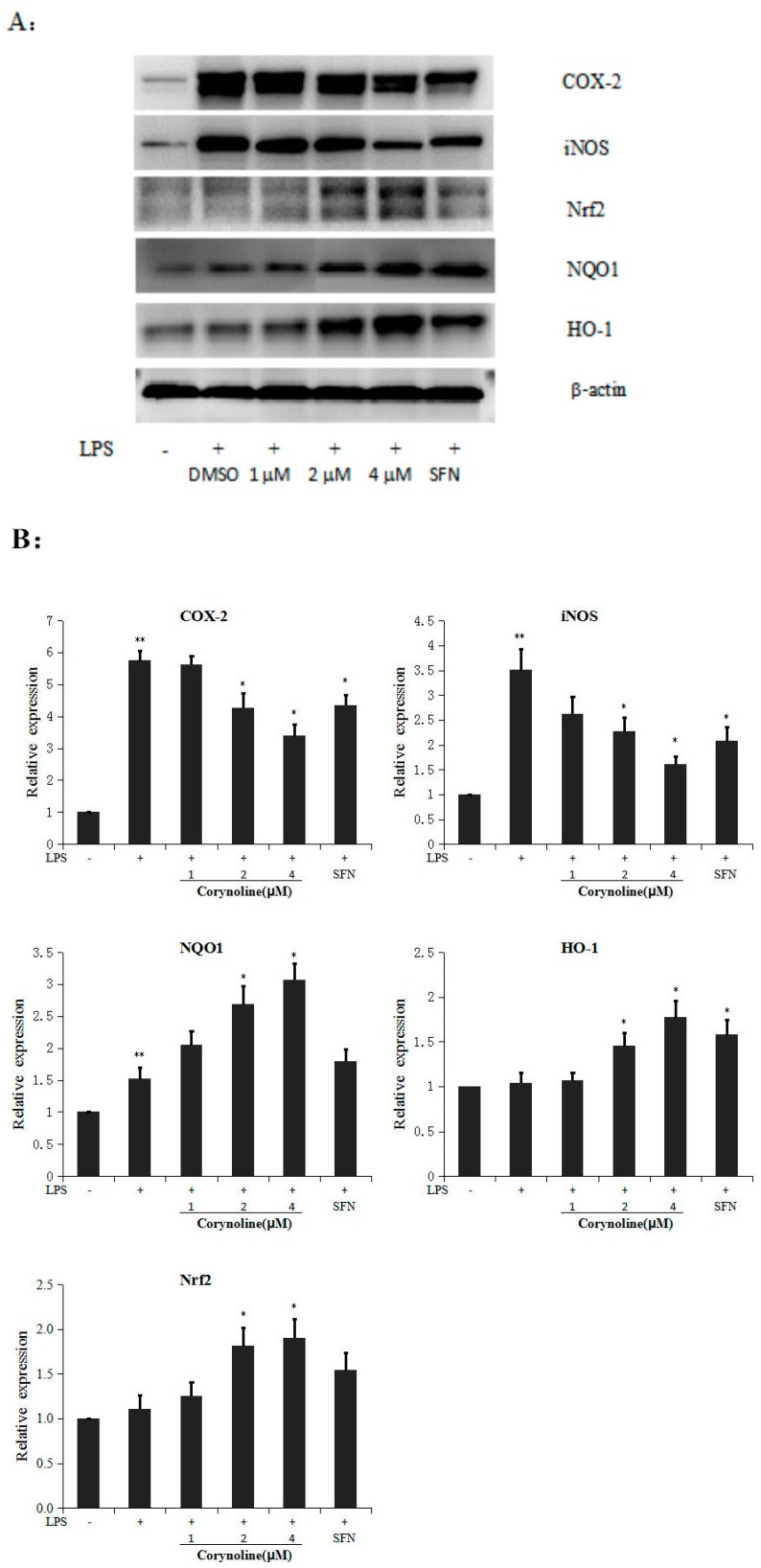
Effect of corynoline on the expression of the iNOS, COX-2, HO-1, NQO1 and Nrf2 proteins in LPS-induced RAW264.7 cells. (**A**) RAW264.7 cells were treated with LPS (150 ng/mL) alone or in combination with corynoline or SFN (each at 1.0 μM). The cells were harvested, and the total protein was extracted at 24 h; the protein levels were measured by Western blotting; (**B**) Corynoline suppressed the LPS-induced expression of iNOS and COX-2 and increased the LPS-induced expression of HO-1, NQO1 and Nrf2. The bands were densitometrically analyzed using ImageJ software (National Institutes of Health, New York, NY, USA, version 1.50a, http://rsbweb.nih.gov/ij). The relative protein expression levels were calculated and compared with those of the control, which were set to 100%. ** *p* < 0.05 compared with the vehicle control. * *p* < 0.05 compared with the LPS-treated group.

Because corynoline showed the strongest induction of ARE-luciferase activity at 24 h (Figure 2B), we evaluated the expression of Nrf2 and its target genes NQO-1 and HO-1 at the mRNA and protein levels. The Nrf2, NQO1 and HO-1 expression levels in LPS-induced RAW264.7 cells were analyzed by quantitative RT-PCR and western blotting to investigate the mechanisms by which corynoline induces the Nrf2/ARE pathway. The relative protein and mRNA expression levels were calculated and compared with the LPS-treated group. The data showed that corynoline dose-dependently increased the expression of Nrf2, HO-1 and NQO1 at the protein and mRNA levels in LPS-induced RAW264.7 cells (Figure 4B and Figure 6C). The results revealed that 4 μM corynoline induced a moderate increase in the expression of the HO-1 protein and a significant increase in the levels of the Nrf2 and NQO-1 proteins (*p* < 0.05) compared with the LPS-treated group. These results indicate that the anti-inflammatory effects of corynoline on the Nrf2/ARE pathway may be mediated by its ability to up-regulate the levels of Nrf2, HO-1 and NQO1.

### 2.4. Knockdown of Nrf2 Decreases Corynoline-Induced Protein Expression of Nrf2 and Nrf2 Target Enzymes

**Figure 5:**
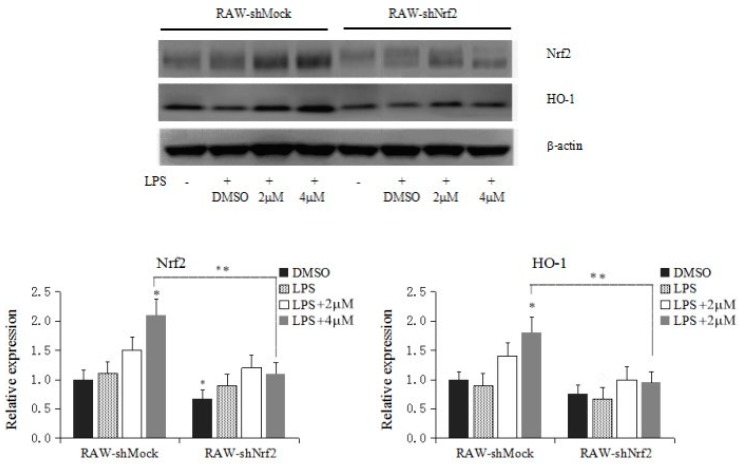
Effect of corynoline on the protein expression of Nrf2 and HO-1 in RAW-shMock and RAW-shNrf2 cells. Cells were incubated with various concentrations of corynoline (2 and 4 μM) for 5 days. The protein levels were measured by protein lysate preparation and western blotting, as described in the Materials and Methods section. The relative expression levels were quantified based on the signal intensity of the corresponding bands from three independent experiments and were normalized using β-actin. The graphical data are presented as the mean SD from three independent experiments. * represent *p* < 0.05, respectively, which indicated significant differences in the target proteins compared with their levels in RAW-shMock cells without corynoline treatment. ** also represent *p* < 0.05, which indicated statistical significance between RAW-shMock and RAW-shNrf2 cells.

The efficiency of short hairpin RNA (shRNA) knockdown was examined, as shown in Figure 5, The protein expression of Nrf2 was significantly decreased after transfection of RAW246.7 cells with shNrf2 in the absence of corynoline treatment (*p* < 0.05). Compared with the RAW-shMock cells, the protein expression of Nrf2 and HO-1 significantly decreased after application of 4 μM corynoline in the RAW-shNrf2 cells. However, application of 4 μM corynoline significantly induced Nrf2 and HO-1 protein expression compared with the control RAW-shMock cells (*p* < 0.05); in contrast, treatment with 2.0 to 4.0 μM corynoline only caused a slight increase in Nrf2 and HO-1 expression in the RAW-shNrf2 cells. These results indicated that corynoline might inhibit inflammatory response in RAW264.7 cells through the upregulation of cellular Nrf2, resulting in an increase in the protein levels of Nrf2 downstream genes, including HO-1.

### 2.5. Corynoline Down-Regulates the Expression of iNOS and COX-2 at the mRNA and Protein Levels

**Figure 6:**
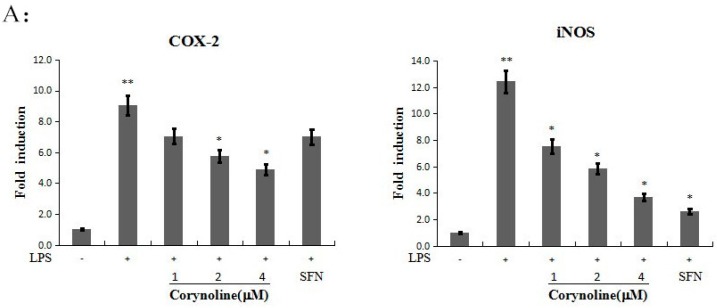

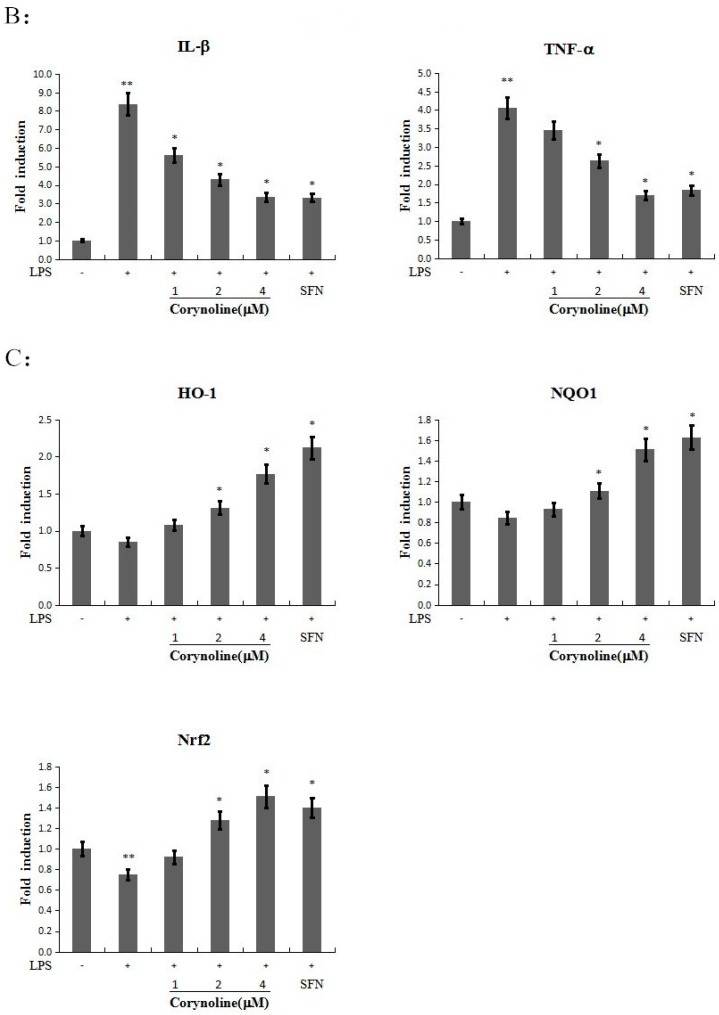
Effect of corynoline on LPS-induced mRNA expression for iNOS, COX-2, IL-1β, TNF-α, HO-1, Nrf2 and NQO1. RAW264.7 cells were treated with LPS (150 ng/mL) either alone or were treated with LPS and corynoline or LPS and SFN (each at 1.0 μM). The cells were harvested for total RNA extraction at 24 h, and the mRNA levels for iNOS, COX-2, IL-1β, TNF-α, HO-1, Nrf-2 and NQO1 were measured using quantitative RT-PCR. Each point represents the mean ± SE; ** *p* < 0.05 as compared to the vehicle control. * *p* < 0.05 as compared to the LPS-treated group. (**A**) Corynoline down-regulates the expression of iNOS, COX-2 at mRNA levels; (**B**) Corynoline down-regulates the expression of 1L-1β,TNF-α at mRNA levels; (**C**) Corynoline up-regulates the expression of HO-1,Nrf-2 and NQO1 in LPS-induced RAW264.7 cells at mRNA levels.

Because corynoline significantly inhibited NO production at 24 h (Figure 3B), we evaluated the expression of iNOS and COX-2 at the mRNA and protein levels. To evaluate the effect of corynoline on the LPS-induced expression of these inflammatory enzymes, the mRNA and protein levels of iNOS and COX-2 in LPS-induced RAW264.7 cells were determined. The relative protein and mRNA expression levels were calculated and compared with those of the LPS-treated group. The data demonstrated that LPS increased both the protein and mRNA expression levels of iNOS and COX-2, whereas corynoline significantly reversed the LPS-induced up-regulation in a dose-dependent manner (Figure 4A and Figure 6A). The results indicated that corynoline inhibited NO production by down-regulating the levels of iNOS and COX-2.

### 2.6. Corynoline Down-Regulates the Expression of IL-1β and TNF-α mRNA and Protein in LPS-Induced RAW264.7 Cells

**Figure 7:**
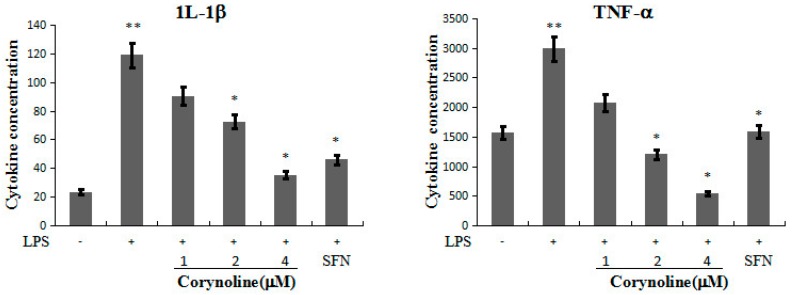
Inhibitory activity of corynoline on LPS–induced protein expression for 1L-1β, TNF-α. RAW264.7 cells were treated with LPS (150 ng/mL) either alone or were treated with LPS and corynoline or LPS and SFN (each at 1.0 μM). The cytokine concentrations were normalized to the protein concentrations. IL-1β and TNF-α levels were measured using an ELISA kit, and the total protein concentrations were measured using a BCA (bicinchoninic acid) protein assay. Each point represents the mean ± SE; ** *p* < 0.05 as compared to the vehicle control. * *p* < 0.05 as compared to the LPS-treated group.

We further evaluated the anti-inflammatory effect of corynoline by testing its effect on pro-inflammatory cytokine (IL-1β and TNF-α) production in the LPS-induced RAW264.7 cells. As shown in Figure 6B, corynoline attenuated the LPS-stimulated mRNA levels of IL-1β and TNF-α in RAW264.7 cells. Accordingly, the ELISA results revealed a significant decrease in the protein levels of IL-1β and TNF-α (*p* < 0.05) in cells that were treated with 4 μg/mL corynoline compared with LPS-treated cells (Figure 7).

### 2.7. Corynoline Inhibits the Phosphorylation of p38 and JNK in LPS-Induced RAW264.7 Cells

**Figure 8:**
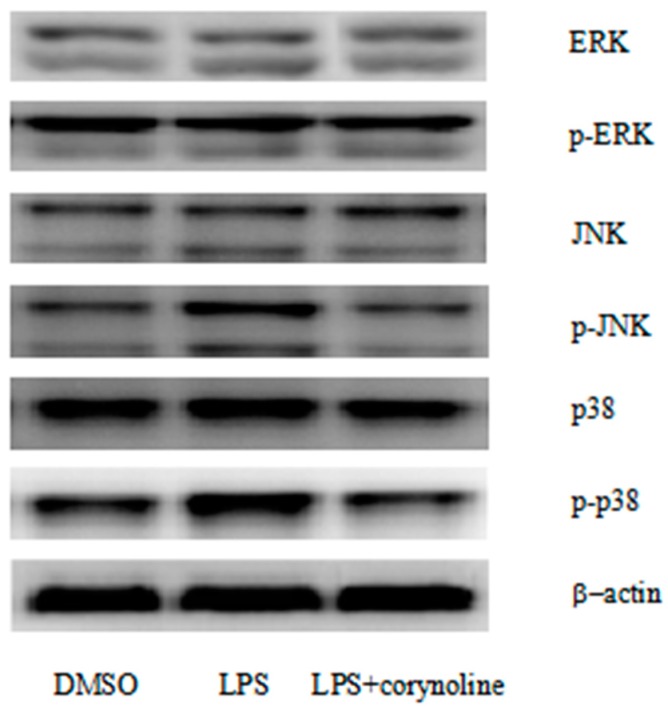
Evaluation of the different cellular signaling pathways affected by corynoline in LPS-induced RAW264.7 cells. Corynoline effectively blocked the activation of p38 MAPK and JNK signaling. RAW264.7 cells were treated with LPS (150 ng/mL) alone or in combination with corynoline (1.0 μM). The cells were harvested, and the proteins were measured after 24 h.

To elucidate the molecular mechanism underlying the anti-inflammatory action of corynoline, we evaluated the MAPKs ERK1/2, JNK and p38. LPS treatment significantly increased the phosphorylation of ERK1/2, JNK and p38, but the total protein levels were not changed. By contrast, the levels of phosphorylated p38 and JNK in the group treated with both corynoline and LPS were significantly reduced. However, the phosphorylation of ERK1/2 by LPS was not reduced by corynoline. These results indicated that corynoline may function as an anti-inflammatory agent by inhibiting the activation of p38 and JNK, but not ERK1/2, in LPS-induced RAW264.7 cells (Figure 8).

## 3. Discussion

Corynoline, an isoquinoline alkaloid, is a major bioactive constituent of *C. bungeana* Turcz. In 2002, Kim reported that corynoline was an acetylcholinesterase inhibitor [27]. Ma and Choi also reported that it exerts fungitoxic and cytotoxic activity [28,29]. Recently, corynoline was found to protect cells from LPS-induced sepsis [30], and it also exhibited concentration-dependent inhibition of cell adhesion [31]. Based on previous reports, we hypothesized that corynoline would demonstrate anti-inflammatory activity. Each step of the underlying mechanism of the anti-inflammation activity of corynoline was also investigated. In preliminary experiments, we investigated inhibition of cell growth by corynoline, acetylcorynoline and protopine in different concentrations. We first set the concentration gradient to 4 to 32 μM. The MTT results revealed that three compounds significantly suppress cell growth in more than 8 μM (data not shown), so the cell toxicity and induction of ARE activity of these compounds was further observed in cells in 1 to 8 μM. Eventually, we find that corynoline antioxidant capacity was the strongest in the three compounds. In addition, corynoline did not affect cell viability in 1 to 4 μM. Therefore, these concentrations were used for the subsequent studies.

NO is thought to play many regulatory roles at each stage of the development of inflammation [29]. NO levels may reflect inflammatory status; thus, NO remains a potential target for the development of therapeutics for inflammatory diseases [32,33]. COX-2 and iNOS are key enzymes involved in NO production [34], and COX-2 and iNOS are also potential targets for assessing potential chemicals that can be used to treat inflammatory diseases. Corynoline dose-dependently reduced the production of nitrite in LPS-induced RAW264.7 cells, which indicated a potential anti-inflammatory effect of corynoline. Furthermore, treatment with 4 μM corynoline significantly decreased the expression of COX-2 and iNOS at both the mRNA and protein levels, which demonstrated an inhibitory effect of corynoline on LPS-induced RAW264.7 cells.

Pro-inflammatory cytokines such as TNF-α and IL-1β can mediate inflammation, and COX-2 and iNOS also play pivotal roles in the inflammatory response to pathologic stimuli [35,36,37]. TNF-α was reported to up-regulate COX-2 expression in the β-amyloid-injected mouse brain [38] and in human NCI-H292 epithelial cells [39]. The overproduction of IL-1β promotes cell/tissue damage during an inflammatory response. Moreover, IL-1β can induce COX-2 and iNOS expression through the p38 MAPK pathway and can mediate NO production in the absence of LPS [40]. In our current study, the data showed that the up-regulated expression of TNF-α and IL-1β in the LPS-induced RAW264.7 cells was reversed by corynoline in a dose-dependent manner. Therefore, corynoline inhibited the inflammatory process and the expression of pro-inflammatory cytokines, such as TNF-α and IL-1β, and down-regulated the expression of pro-inflammatory genes such as COX-2 and iNOS.

Nrf2 is a cytoprotective transcription factor that induces the expression of several antioxidant/detoxifying enzymes [41,42]. Because oxidative stress is closely related to inflammatory processes, Nrf2 plays an important role in the regulation of inflammation [17,18]. It was reported that many chemopreventive compounds possess antioxidant and anti-inflammatory activities that involve the Nrf2/ARE pathway [43]. Moreover, Nrf2 plays a critical role in regulating the expression of COX-2 and iNOS [18]. Interestingly, it was reported that there was no significant decrease in the expression of anti-inflammatory genes and an increase in HO-1 expression in Nrf2−/− macrophages treated with either PEITC or CUR, but there was a significant decrease in the expression of COX-2 protein and an increase in the expression of HO-1 in Nrf2+/+ macrophages treated with PEITC compared with those treated with CUR. These results showed that the anti-inflammatory effect was attenuated in the primary Nrf2−/− peritoneal macrophages [44,45]. Therefore, we examined the effects of corynoline on ARE-luciferase-transfected HepG2 C8 cells to investigate whether corynoline induces Nrf2/ARE activity. Corynoline significantly induced Nrf2/ARE activity, indicating that the Nrf2/ARE pathway may be involved in the anti-inflammatory activity of corynoline. Nrf2 can rapidly respond to oxidants by stimulating the transcriptional activation of detoxification genes, such as NQO1 and HO-1 [46,47]. HO-1 has been reported to mediate antioxidant and anti-inflammatory effects both in vitro and in vivo [48,49]. In our study, corynoline treatment significantly increased the expression of NQO1 and HO-1 at both the mRNA and protein levels, indicating that corynoline may protect cells from inflammation by activating the Nrf2/ARE pathway and inducing the expression of NQO1 and HO-1. In order to further research that corynoline may protect cells from inflammation by activating the Nrf2/ARE pathway, the critical role of Nrf2 induction after corynoline treatment in RAW264.7 cells was investigated using a stable Nrf2-knockdown cell line. Compared with that in the RAW-shMock cells, the result showed that the protein expression of Nrf2 and HO-1 significantly decreased after application of corynoline in the RAW-shNrf2 cells. It is concluded that corynoline inhibit inflammatory response cells through activating the Nrf2/ARE pathway.

MAPKs, a group of serine/threonine kinases including ERK1/2, p38 and JNK, play prominent roles in regulating a wide range of physiological processes [14]. MAPKs are activated in response to various extracellular stimuli and mediate signal transduction from the cell surface to the nucleus. ERK contributes to cell division, proliferation, differentiation, and survival and can be used as a target for screening anticancer agents [14,50]. In general, p38 is related to diseases, such as asthma and autoimmunity, and can be activated in response to inflammatory cytokines, growth factors, and Ultraviolet (UV) light [14,51]. JNK, a critical regulator of transcription, is also activated by UV radiation and inflammatory cytokines [14,51]. Moreover, MAPKs have been shown to be involved in the transcriptional regulation of pro-inflammatory mediators, such as iNOS, COX-2 and TNF-α, in response to LPS stimulation. JNK was reported to modulate the expression of iNOS in LPS-induced RAW264.7 cells [14], whereas p38 regulates the expression of COX-2 [50]. Based on previous observations, the inhibition of p38 and JNK MAPK plays an important role in regulating the transcription of COX-2, iNOS and pro-inflammatory cytokines during inflammatory processes. In the present study, we examined the effects of corynoline on these MAPKs, and found that corynoline inhibited the phosphorylation of p38 and JNK, but not ERK1/2. These results suggest that the phosphorylation of p38 and JNK may be involved in the anti-inflammatory actions of corynoline. Recent evidence demonstrated that Nrf2 regulates anti-inflammatory reactions through the MAPK pathway [52,53]. Kong et al. have demonstrated that MAPK is involved in ARE activation that is driven by Nrf2-dependent activation of MAPK [54]. The Nrf2-regulated MAPK pathway may be a potential mechanism for the anti-inflammatory effects of corynoline.

## 4. Materials and Methods

### 4.1. Chemicals and Reagents

Dimethyl sulfoxide (DMSO) and sulforaphane (SFN) were purchased from LKT Laboratories (St. Paul, MN, USA). Dulbecco’s Modified Eagle’s Medium (DMEM) and fetal bovine serum (FBS) were obtained from Invitrogen-Gibco (Grand Island, NY, USA). The anti-COX2, anti-iNOS, anti-p38, anti-ERK, anti-JNK, anti-actin, anti-HO1, anti-NQO1 and anti-Akt primary antibodies and secondary antibodies were purchased from Santa Cruz Biotechnology (Santa Cruz, CA, USA). The anti-phospho-JNK, anti-phospho-ERK, anti-phospho-p38, anti-Nrf2 and anti-phospho-Akt primary antibodies were acquired from Cell Signaling Technology (Danvers, MA, USA). All other chemicals and reagents were purchased from Sigma-Aldrich (St. Louis, MO, USA).

### 4.2. Extraction and Isolation of Alkaloids

Ten kilograms of dried *C. bungeana* was extracted under reflux with 80 L of ethanol–water (95:5, *v*/*v*) two times for 2 h each time and then filtered. The combined filtrate was concentrated under vacuum to obtain the crude extract (700 g). Next, the crude extract was dissolved in water and subjected to chromatography on a D-101 macroporous resin column at 60 °C to give fractions (water→30% ethanol→60% ethanol→95% ethanol).The 95% ethanol fraction (50.3 g) was subjected to silica gel column chromatography (9 × 120 cm, 1 kg) and then eluted with CHCl_3_/MeOH (30:1→20:1→10:1→5:1→2:1→1:1) to give 186 fractions (Frs.1–186). Fractions 5–10 (6.07 g) were repeatedly chromatographed on silica gel and Sephadex LH-20 to yield corynoline (900 mg) and acetylcorynoline (120 mg). Fractions 15–18 (1.01 g) were also repeatedly chromatographed on silica gel and Sephadex LH-20 to give protopine (50.5 mg) (Figure 1). The structures of these compounds were identified by comparing their ^1^H-, ^13^C-NMR and ESI-MS spectra with those previously reported in the literature [27]. The compounds used in this study were checked by HPLC and exhibited >97% purity [55].

### 4.3. Cell Culture and Treatments

Human hepatoma HepG2 cells were purchased from American Type Culture Collection (ATCC, Rockville, MD, USA). The HepG2-C8 cell line was established in Dr. Ah-Ng Tony Kong’s laboratory by transfecting the HepG2 cells with a pARE-T1-luciferase construct (kindly provided by Dr. William Fahl, University of Wisconsin) using the FuGENE 6 method, as previously described [28]. The cells were routinely cultured in DMEM supplemented with 10% FBS, 1.17 g/L sodium bicarbonate, 100 units/mL penicillin and 100 μg/mL streptomycin and incubated at 37 °C in a humidified atmosphere containing 5% CO_2_. The cells were grown to 80% confluence, split by detaching them with trypsin, and then sub-cultured in fresh medium three times per week after washing with Versene (Gibco, Carlsbad, CA, USA) [16].

The RAW264.7 murine macrophage cell line was obtained from ATCC. The cells were maintained in DMEM supplemented with 10% FBS (and 1% penicillin/streptomycin) at 37 °C and 5% CO_2_. RAW246.7 cells stably transfected with shMock and shNrf2-knockdown were maintained in DMEM supplemented with 5% FBS. RAW264.7 cells were treated with *E. coli* LPS (150 ng/mL) in related experiments. The cells were treated with corynoline (1, 2 or 4 μM) or SFN (5 µM) either alone or in combination with LPS for different time intervals, unless otherwise specified. The cells were treated with 0.05% DMSO as a vehicle control [16].

### 4.4. Cell Viability Assay

The cytotoxicity of corynoline was tested in the RAW264.7 murine macrophages and HepG2-C8 cells using the CellTiter 96 aqueous nonradioactive cell proliferation assay reagent (MTS) [3-(4,5-dimethylthiazol-2-yl)-5-(3-carboxymethoxyphenyl)-2-(4-sulfophenyl)-2H-tetrazolium, inner salt; MTS] (Promega, Madison, WI, USA). After 24 h of cell culture in 96-well plates, the cells were treated with various concentrations of corynoline for an additional 24 h. The cells were then incubated with MTS for 1 h at 37 °C. Absorbance at 490 nm was measured using a μQuant Biomolecular Spectrophotometer (Bio-Tek Instruments, Winooski, VT, USA) [13].

### 4.5. Evaluation of ARE Reporter Gene Activity by Luciferase Assay

HepG2-C8 cells stably expressing the ARE luciferase reporter were cultured in 12-well plates. The cells were treated with corynoline for 24 h, and luciferase activity was measured using a Promega luciferase kit. Using a slight modification of the manufacturer’s protocol, after 24 h of treatment, the cells were washed twice with ice-cold PBS and harvested using 1× reporter lysis buffer. The cell lysate was centrifuged at 9660× *g* for 5 min at 4 °C, and the supernatant was used for the luciferase activity assay. Luciferase activity was measured using a SIRIUS luminometer (Berthold Detection System GmbH, Pforzheim, Germany). After normalization to the protein concentration, luciferase activity was described as the fold induction of the samples compared with vehicle control-treated cells [13].

### 4.6. Evaluation of the Increase in NO Production Using the Nitrite Assay

The MTS assay was used to evaluate any potential toxicity, as described above. Nitrite accumulation in the culture media was used as an indicator of NO production. The cells were cultured in 96-well culture plates for 24 h and stimulated with LPS (150 ng/mL) in the presence or absence of corynoline, acetylcorynoline and protopine for an additional 24 h. The controls were 0.1% DMSO with and without LPS. After isolating the supernatant fractions, equal volumes of Griess reagent (1% sulfanilamide, 0.1% naphthylethylenediamine dihydrochloride, and 2% phosphoric acid) were added to the cells and incubated for 10 min at room temperature. Nitrite production was measured using a μQuant Biomolecular Spectrophotometer (Bio-Tek Instruments) at an absorbance of 540 nm. The results are expressed as the concentration of nitrite produced [43].

### 4.7. Protein Lysate Preparation and Western Blotting

All cells were harvested in radioimmunoprecipitation assay (RIPA) buffer containing a protein inhibitor cocktail (Sigma, St. Louis, MO, USA). The bicinchoninic acid (BCA) method (Pierce, Rockford, IL, USA) was used to determine the protein concentrations of the cell lysates. Equal amounts of (20 μg) the total protein from each sample were resolved by 4%–15% sodium dodecyl sulfate (SDS)-polyacrylamide gel electrophoresis (Bio-Rad, Hercules, CA, USA) and electrotransferred to a polyvinylidene difluoride (PVDF) membrane (Millipore, Bedford, MA, USA). After blocking with 5% bovine serum albumin (BSA; Fisher Scientific, Pittsburgh, PA, USA), the PVDF membrane was sequentially probed with specific primary antibodies and HRP-conjugated secondary antibodies. The blots were then visualized using the SuperSignal enhanced chemiluminescence detection system and recorded using a Gel Documentation 2000 system (Bio-Rad) [18].

### 4.8. Quantitative Real-Time Polymerase Chain Reaction (qPCR)

Total RNA was extracted from treated RAW264.7 macrophages using the RNeasy Mini Kit (QIAGEN, Valencia, CA, USA). First-strand cDNAs were synthesized from 1 μg of total RNA using the SuperScript III First-Strand Synthesis System for RT-PCR (Invitrogen, Carlsbad, CA, USA), according to the manufacturer’s instructions. The cDNAs were used as the template for PCR reactions performed on the ABI7900HT system (Life Technologies, Grand Island, NY, USA). The mRNA quantification was carried out using ∆∆CT method; relative fold change from treatment group was normalized to the vehicle control group whereas GAPDH was set as internal reference. The sequences of the primers for inflammation-related genes are listed in Table 1 [43].

### 4.9. Cytokine Measurements

RAW264.7 cells were treated with SFN (5 µM), corynoline (1, 2, or 4 µM) or LPS (150 ng/mL), as described above. The cells were then collected, and the cytokine concentrations were determined using ELISA kits from Invitrogen and R&D Systems. The IL-1β and TNF-α ELISAs were performed according to the manufacturers’ instructions. The cytokine concentrations were normalized to the protein concentrations, which were determined using a BCA protein assay (Pierce). The data were obtained from three independent experiments and were expressed as the fold induction compared with the vehicle control (cells incubated in medium containing 0.1% DMSO) [46].

### 4.10. Statistical Analyses

Data are presented as the mean ± SE for the indicated number of independently performed experiments. Statistical analyses were performed using one-way ANOVA, followed by Dunnett’s test. At least three independent experiments for each assay were conducted. Tests of zero correlation were used to determine significant correlations. In both analyses, *p* < 0.05 was used to denote significance.

## 5. Conclusions

Our study shows that corynoline acts as an anti-inflammatory agent. Corynoline treatment inhibits the overproduction of NO, TNF-, IL-6 and IL-1β and the over-expression of iNOS and COX-2 in LPS-induced RAW264.7 cells. The inhibitory action of corynoline is partly mediated by the suppression of JNK and p38 phosphorylation, but not ERK1/2 phosphorylation; these phosphorylation events are regulated by the Nrf2/ARE pathway. These findings provide a partial molecular explanation for the anti-inflammatory properties of corynoline.

## Figures and Tables

**Table 1:** Murine primers for PCR.

Gene	Forward	Reward
GAPDH	5′-TGC TCG AGA TGT CAT GAA GG-3′	5′-TGG CGC TCA TCG TAG GCT TT-3′
COX-2	5′-TCC TCC TGG AAC ATG GAC TC-3′	5′-TGA TGG TGG CTG TTT TGG TA-3′
iNOS	5′-GTG GTG ACA AGC ACA TTT GG-3′	5′-GGC TGG ACT TTT CAC TCT GC-3′
HO-1	5′-GCT CGA ATG AAC ACT CTG GAG AT-3′	5′-TCC AGA GAG AAA GGA AAC ACA GG-3′
NQO1	5′-CAG AAA TGA CAT CAC AGG TGA GC-3′	5′-CTA AGA CCT GGA AGC CAC AGA AA-3′
Nrf2	5′-GGC AGA GAC ATT CCC ATT TGT AG-3′	5′-TCG CCA AAA TCT GTG TTT AAG GT-3′
TNF-α	5′-ACG GCA TGG ATC TCA AAG AC-3′	5′-GGT CAC TGT CCC AGC TT-3′
IL-1β	5-′GAG TGT GGA TCC CAA GCA AT-3′	5′-CTC AGT GCA GGC TAT GCT TT-3′
